# Impact of GAD65 and IA2 autoantibodies on islet allograft survival

**DOI:** 10.3389/fcdhc.2023.1269758

**Published:** 2023-11-13

**Authors:** Joana R. N. Lemos, Raffaella Poggioli, Jonathan Ambut, Nujen C. Bozkurt, Ana M. Alvarez, Nathalia Padilla, Francesco Vendrame, Camillo Ricordi, David A. Baidal, Rodolfo Alejandro

**Affiliations:** ^1^ Diabetes Research Institute (DRI) and Clinical Cell Transplant Program, University of Miami Miller School of Medicine, Miami, FL, United States; ^2^ Division of Endocrinology, Diabetes and Metabolism, Department of Medicine, University of Miami Miller School of Medicine, Miami, FL, United States; ^3^ Division of Cellular Transplantation, Department of Surgery, University of Miami Miller School of Medicine, Miami, FL, United States

**Keywords:** islet transplantation, autoantibodies, type 1 diabetes, GAD65 autoantibody, IA2 autoantibody, allograft survival

## Abstract

**Introduction:**

Islet transplantation (ITx) shows promise in treating T1D, but the role of islet autoantibodies on graft survival has not been clearly elucidated. We aimed to analyze the effect of GAD65 and IA2 autoantibody status on graft survival and attainment of insulin independence in subjects with T1D who underwent ITx.

**Method:**

We conducted a retrospective cohort study on 47 ITx recipients from 2000 to 2018. Islet infusion was performed via intrahepatic portal (n=44) or onto the omentum via laparoscopic approach (n=3). Immunosuppression involved anti-IL2 receptor antibody, anti-TNF, and dual combinations of sirolimus, tacrolimus, or mycophenolate mofetil (Edmonton-like) in 38 subjects (80.9%). T-cell depletion induction with Edmonton-like maintenance was used in 9 subjects (19%). GAD65 and IA2 autoantibodies were assessed pre-transplant and post-transplant (monthly) until graft failure, and categorized as persistently negative, persistently positive, or seroconverters. Graft survival was analyzed using U-Mann-Whitney test, and Quade’s nonparametric ANCOVA adjusted for confounders. Kaplan-Meier and Log-Rank tests were employed to analyze attainment of insulin independence. P value <0.05 indicated statistical significance.

**Results:**

ITx recipients with persistent autoantibody negativity (n = 21) showed longer graft function (98 [61 – 182] months) than those with persistent autoantibody positivity (n = 18; 38 [13 – 163] months), even after adjusting for immunosuppressive induction protocol (P = 0.027). Seroconverters (n=8) had a median graft survival time of 73 (7.7 – 167) months, which did not significantly differ from the other 2 groups. Subjects with persistently single antibody positivity to GAD65 (n = 8) had shorter graft survival compared to negative islet autoantibody (GAD65/IA2) subjects (n = 21; P = 0.016). Time of graft survival did not differ in subjects with single antibody positivity to IA2. The proportion of insulin independence attainment was similar irrespective of autoantibody status.

**Conclusion:**

The persistence of islet autoantibodies, as markers of islet autoimmunity, may represent an underappreciated contributing factor to the failure of transplanted β cells. Whether induction with T-cell depletion may lead to improved graft survival, independent of islet autoantibody status, could not be evaluated in our cohort. Larger prospective studies are needed to further address the role of islet autoantibody status on islet graft survival.

## Introduction

Type 1 diabetes (T1D) is a chronic autoimmune disorder in which self-reactive cytotoxic T-cells gradually destroy the insulin producing β-cell in the pancreatic islets requiring life-long insulin treatment. Therapies aimed at restoring β-cell mass resulting in sustained metabolic control in the absence of insulin therapy are currently being evaluated in clinical trials. Islet transplantation has been confirmed to be a highly promising therapy for patients with T1D due to its ability to achieve near normal glycemia, restore hypoglycemic awareness, and prevent episodes of severe hypoglycemia (1). While islet transplantation is considered a safe procedure, it is important to note that achieving long-lasting insulin independence and ensuring long-term graft survival remain ongoing challenges. Success of β-cell replacement strategies not only requires prevention of allograft rejection, but also recurrence of autoimmunity, as has been demonstrated to occur in experimental models (2, 3).

Over the years, fundamental improvements have been achieved in protecting the transplanted islets from inflammation and allo-rejection, as well as in the management of complications related to immunosuppression. Nevertheless, a large number of islet transplants fail over time, for reasons not entirely known. The genetic predisposition to T1D has been well characterized, but environmental factors, such as viral infections, also contribute to the initiation of the inflammation that follows the autoimmune diabetes progression, ultimately resulting in β cell death (4, 5). Autoantibodies play a role in T1D, as in other autoimmune diseases (6, 7) The major islet- autoantibodies are against insulin (IA), L-Glutamic acid decarboxylase 65 (GAD65), Protein tyrosine phosphatase like protein (IA-2), and the β -cell zinc transporter (ZnT8) (8, 9).

However, the prognostic role of autoantibodies in the setting of islet transplantation is controversial. While the presence of autoantibodies may just represent a surrogate marker of autoimmunity, it is known that the risk of progression to T1D is predicted by the number of serum islet autoantibodies (4, 10). For this reason, there is a growing interest in understanding if changes in islet autoantibody status can represent an early marker of islet graft dysfunction due to recurrence of autoimmune diabetes.

Transplanting islets into patients with T1D re-exposes them to islet autoantigens. While immunosuppression is successful in preventing rejection of the transplanted islets, it remains uncertain whether it can also prevent the reoccurrence of autoimmunity. The reappearance of autoimmunity in T1D has been observed following pancreas transplantation, irrespective of HLA compatibility (11, 12). Despite this observation, there is a lack of effective screening markers to identify patients who are at risk of experiencing this recurrence.

Considering the conflicting evidences of the role of islet autoantibodies in islet transplantation outcomes, we aimed to analyze the effect of GAD65 and IA2 autoantibody status on graft survival and attainment of insulin independence in subjects with T1D who underwent islet transplantation.

## Materials and methods

### Study population

A retrospective cohort study of 47 ITx recipients with T1D (islet alone, n=40; and islet after kidney, n=7) was conducted from 2000 to 2018 at the Clinical Cell Transplant Program, Diabetes Research Institute, University of Miami. Out of the 47 subjects, 44 were transplanted exclusively via percutaneous intrahepatic portal infusion and 3 subjects were transplanted via a laparoscopic approach onto the omentum surface. Islet allograft failure was defined as a stimulated C-peptide <0.3 ng/mL following a mixed meal tolerance test (13). Study procedures were reviewed and approved by the University of Miami Institutional Review Board. All participants provided written informed consent and were enrolled in different protocols (ClinicalTrials.gov Identifier: NCT00213003, NCT00306098, NCT01999374, NCT00315588, NCT 00315614, and NCT00315627).

### Immunosuppression

At total of 38 subjects (80.9%) underwent Edmonton-like induction immunosuppression with anti-IL2 (daclizumab or basiliximab) plus anti-TNF (infliximab or etanercept), whereas 9 subjects (19.1%) received induction with a T-cell depleting agent (thymoglobulin or alemtuzumab) (13–16). Maintenance immunosuppression consisted of a dual combination strategy with either sirolimus, tacrolimus or mycophenolate mofetil (MMF). Target trough levels for sirolimus were 12-15 ng/mL for 3 months, then 7-10 ng/mL thereafter; tacrolimus trough levels were kept at 3-6 ng/mL (or 8-10 ng/mL if subject was on tacrolimus-MMF). MMF target dose was 1000 mg PO twice daily with the dose adjusted depending on subject tolerance.

### Antibodies detection and categorization

Autoantibodies anti-GAD65 and IA2 were analyzed pre-transplant and monthly during the post-transplant period up to graft failure and categorized as follows: persistently negative (GAD65 and IA2), persistently positive (GAD65 and/or IA2), and seroconverters (GAD65 or IA2). Seroconverters were subjects who were either negative for islet autoantibodies pre-ITx and developed persistent antibody positivity post-ITx (to any antibody) or who were single-antibody positive pre-ITx and developed a second persistently positive antibody post-ITx.

Autoantibodies to 65-kilodalton isoform of GAD65 and IA-2 were measured using radio-immunoassays validated in the proficiency workshops of the Immunology of Diabetes Society and Centers for Disease Control and Prevention (12, 17). Autoantibody levels are expressed as the ratio of the autoantibody index levels of the patient over the cut-off index of each assay. A ratio of ≥1 indicates a positive result.

### Histocompatibility

HLA typing was performed, and calculations were based on the level of HLA-A, -B, and -DR antigen specificities.

### Statistical analysis

Results are expressed as mean ± standard deviation (SD), median (25th percentile; 75th percentile) or percentages. Descriptive, clinical, and transplant characteristics between subjects were analyzed through unpaired two tailed T-test with Welch correction, U Mann-Whitney, or chi-square test, as appropriate. Analysis of median of graft survival between groups was done by U-Mann-Whitney test. Quade’s nonparametric ANCOVA was used considering Rank of the dependent variable, in order to evaluate the time of graft function according to GAD65 and/or IA2 antibodies positivity, adjusting for induction immunosuppression. To assess the impact of autoantibody status and time on fasting and stimulated glucose and C-peptide levels, we used Generalized Estimated Equation (GEE) models. We specified separate models for each outcome variable (fasting glucose, stimulated glucose, fasting C-peptide, stimulated C-peptide). The GEE models take into account the within-subject correlation and allowed us to account for the repeated measurements over time. Analyses of gain of insulin independence were performed through Kaplan-Meier curves, and Log-Rank tests. Data were analyzed using IBM SPSS^®^ v28.0 (New York, USA). A P value <0.05 was considered to indicate statistical significance.

## Results

Subjects were aged 42.8 ± 8.5 years at the time of ITx, and 61.7% (n= 29) were female. Mean body mass index of the recipients was 24.4 ± 3.3 kg/m^2^, and diabetes duration 29.4 ± 11.5 years. Subjects were followed for 66.9 (24.9 – 171.6) months. Forty percent of the subjects received a second infusion (n = 19), and median time between first and second infusion was 51.5 (35 – 106) days. Nine patients (19%) received T-cell depletion, and 38 (81%) received Edmonton protocol as the induction immunosuppressive agent.

In our cohort, 36.2% of subjects developed graft failure (n=17), 31.9% subjects with graft function withdrew consent and discontinued immunosuppression due to medical complications (n =15), and 31.9% subjects had persistent graft function throughout the study follow-up (n = 15). We also examined the proportion of patients that were seropositive for CMV among the 3 groups, due to CMV infection’s potential role in the development of T1D autoimmunity and its potential role in graft disfunction.

Out of the 47 patients, 21 were persistently autoantibody negative, 13 were persistently single antibody positive (8 GAD65 positive and 5 IA2 positive), 5 persistently double antibody positive, and 8 positive seroconverters (6 GAD65 and 2 IA2 antibody conversions). Out of 8 seroconverters, 7 (87.5%) received Edmonton immunosuppressive protocol (induction with anti-IL2 receptor) and 1 (12.5%) received T-cell depletion. The median time from the first infusion until the seroconversion was 18 (5 – 33) days. A range from 1 to 3 samples were evaluated for the presence of autoantibodies during the pre-transplant period, and 47.1 ± 31.4 samples during the post-transplant period. The mean number of days between samples taken was 98.3 ± 74.4 during the pretransplant period, and 48.4 ± 30 during the posttransplant period.

Clinical and transplant characteristics of subjects according to autoantibodies status are described in **Table 1**.

**Table 1:** Clinical and transplant characteristics according to GAD65 and/or IA2 autoantibodies positivity.

	GAD65/IA2 negative (n = 21)	GAD65/IA2 positive (n = 18)	GAD65/IA2 seroconverters (n = 8)	P- value
**Recipient age at ITx, years (**± SD)	45 ± 8	43 ± 9	37 ± 7	0.058
**Female, n (%)**	10 (47.6)	12 (66.7)	7 (87.5)	0.103
**BMI at ITx, kg/m^2^ (**± SD)	24.8 ± 3.88	23.0 ± 2.12	25 ± 3	0.205
**Median duration of diabetes, years (25^th^ – 75^th^)**	35 (27 – 39)	28 (14 – 39)	26 (14 – 32)	0.010
**HbA1C pre-ITx, % (**± SD)	7.19 ± 1.27	7.48 ± 1.15	7.59 ± 1.13	0.644
**Islet equivalents infused per kg (1,000; 25^th^ – 75^th^)**	12.01 (8.4 – 16.4)	12.56 (9.5 – 13.3)	13.3 (10.3 – 20.2)	0.763
**Immunosuppression induction Edmonton like protocol, n (%)**	15 (71.4)	16 (88.9)	7 (87.5)	0.334
**Immunosuppression induction T-cell depletion protocol, n (%)**	6 (28.6)	2 (11.1)	1 (12.5)	0.334
**HLA-DR3,**	2 (9.5)	3 (16.7)	0	0.434
**HLA-DR4**	12 (57.1)	10 (55.6)	8 (100)	0.065
**HLA-A24**	3 (14.3)	0	2 (25)	0.124
**CMV infection, n (%)**	10 (47.6)	8 (44.4)	3 (37.5)	0.886

**Figure 1:**
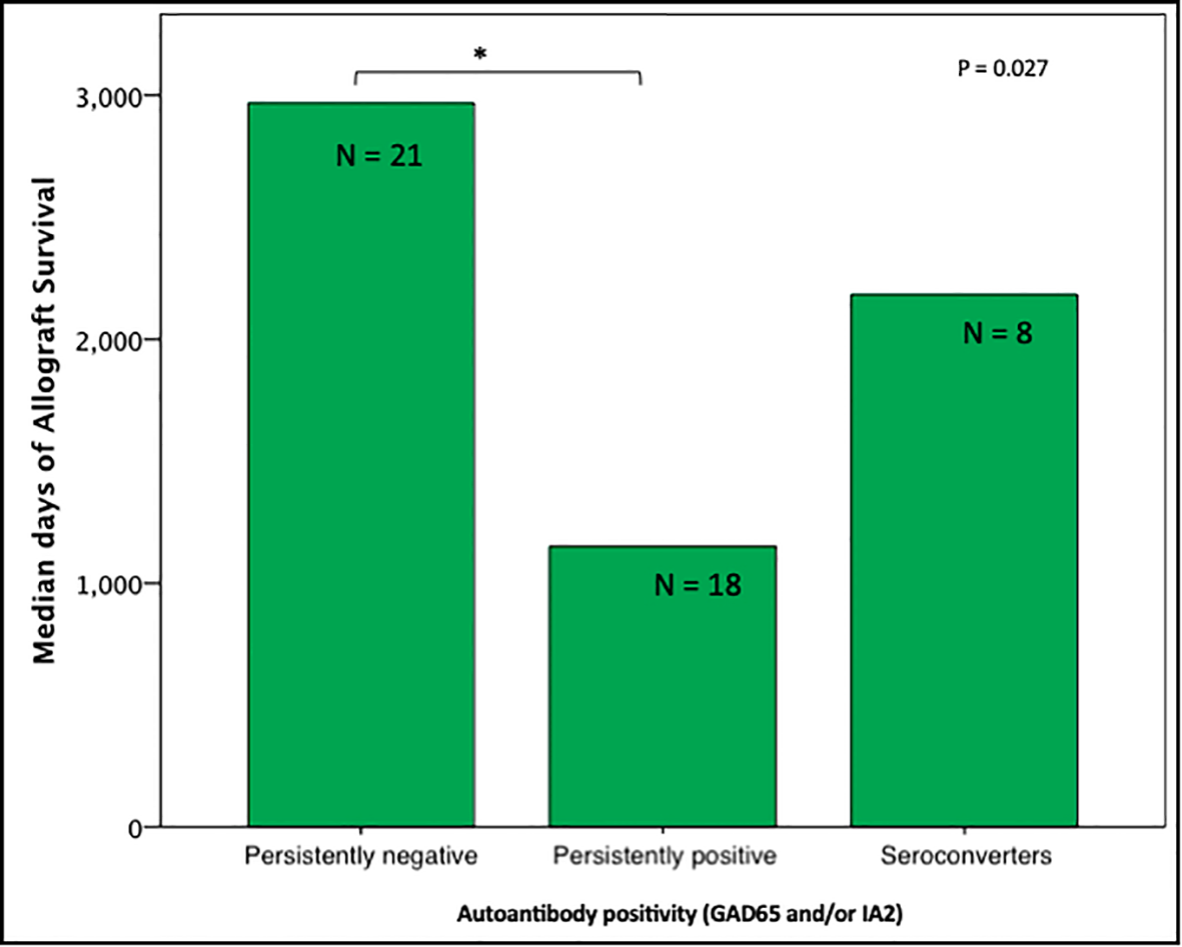
Median days of graft function considering positivity, negativity, or seroconvertion for GAD65/IA2 antibodies (n=47); Persistently positive = positivity for 1 or 2 autoantibodies. *P < 0.05 is noted between subjects persistently positive and subjects persistently negative.

Unadjusted analyses for graft survival showed that persistently autoantibodies negative (GAD65 and/or IA2) recipients (n=21) had longer graft function (98 [61 – 182] months) compared to persistently autoantibody positive recipients (n=18; 13 [38 – 163] months; P = 0.030). Seroconverters (n=8) had a median time of graft survival of 73 (7.7 – 167) months (**Figure 1**) which was not significantly different when compared to the other 2 groups.

**Figure 2:**
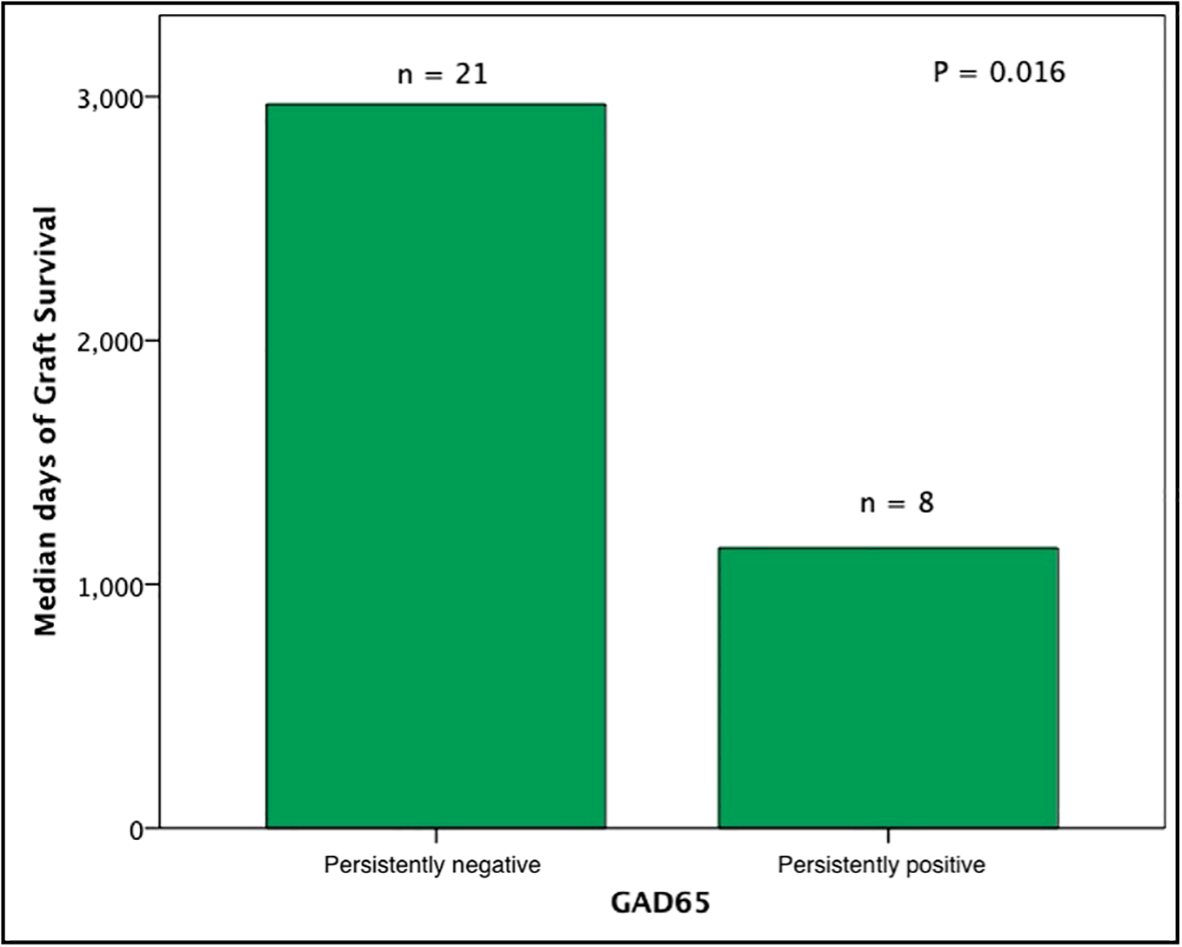
Median days of graft function considering positivity or negativity for GAD65 antibodies only. In this analysis subjects seroconverters and subjects positive for IA2 were excluded (n = 29).

**Figure 3:**
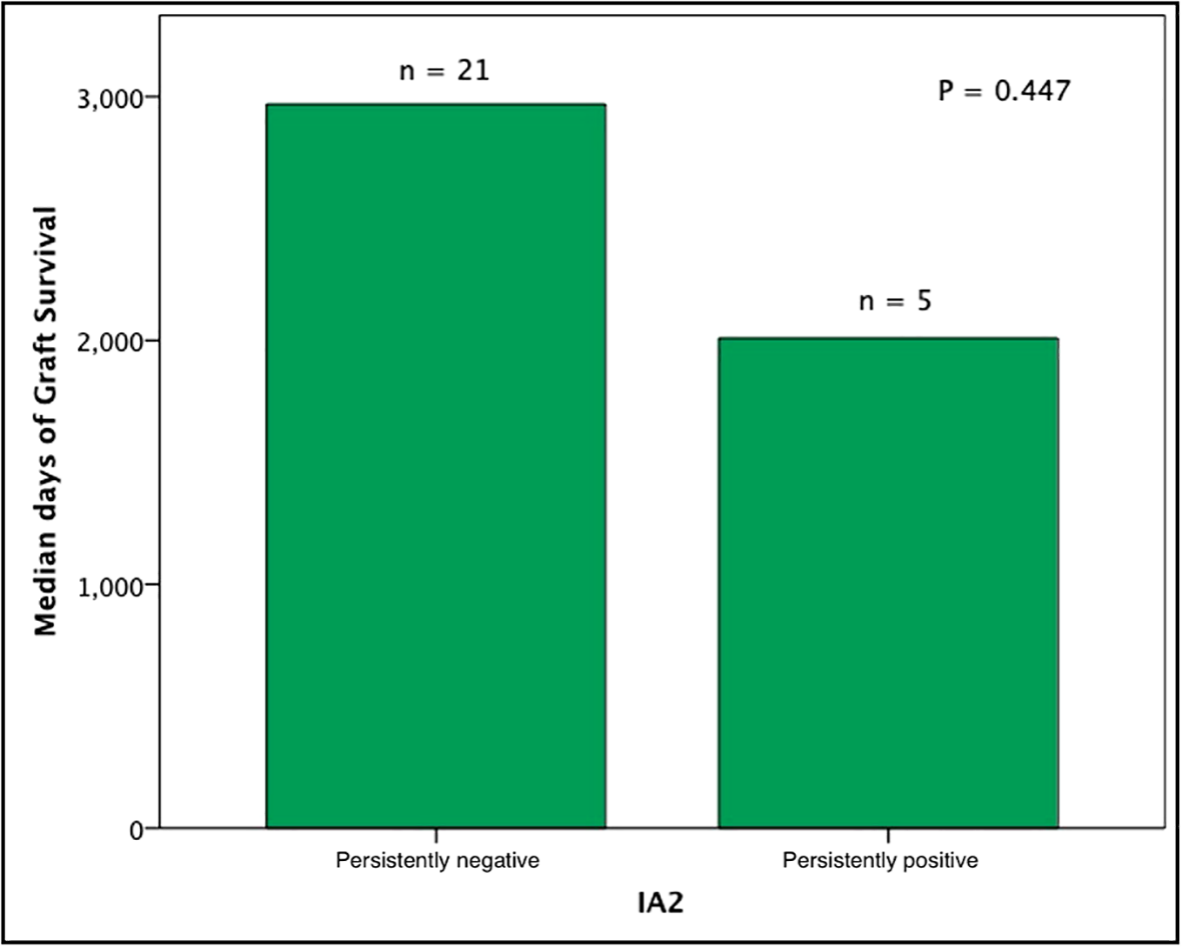
Median days of graft function considering positivity or negativity for IA2 antibodies only. In this analysis, subjects seroconverters and subjects positive for GAD65 were excluded (n = 26).

**Figure 4:**
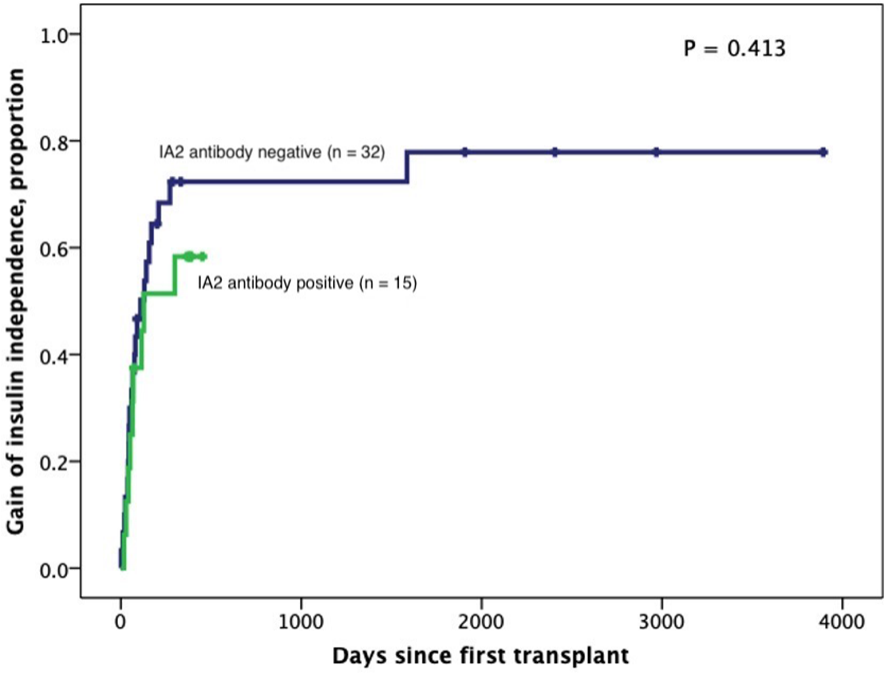
Kaplan-Meier curves of gain of insulin independence according to IA2 positivity (n=47). Patients were censored at the time they achieved insulin independence. Log-Rank test was performed for analysis between groups.

**Figure 5:**
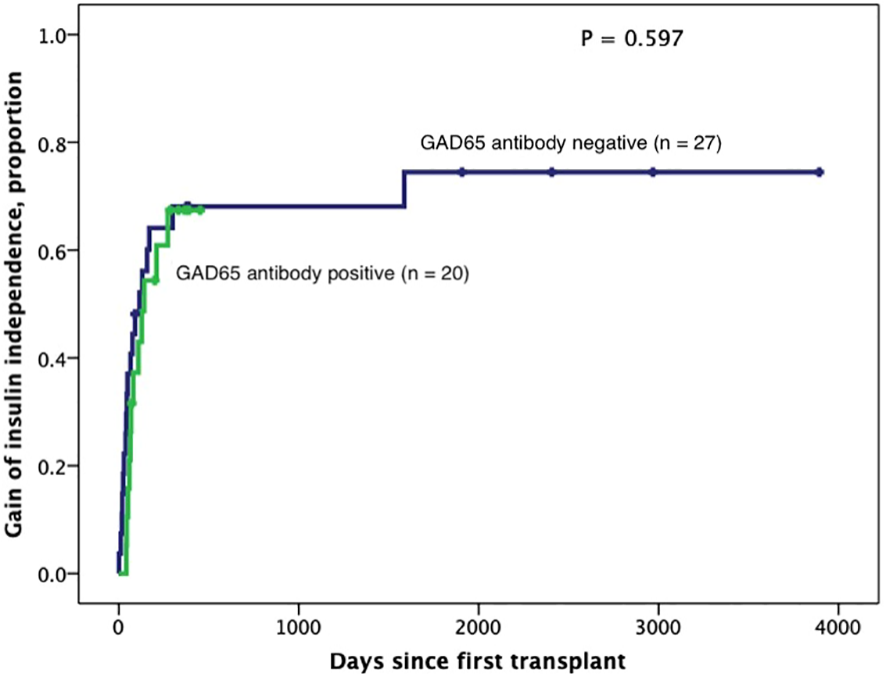
Kaplan-Meier curves of gain of insulin independence according to GAD65 positivity (n=47). Patients were censored at the time they achieved insulin independence. Log-Rank test was performed for analysis between groups.

**Figure 6:**
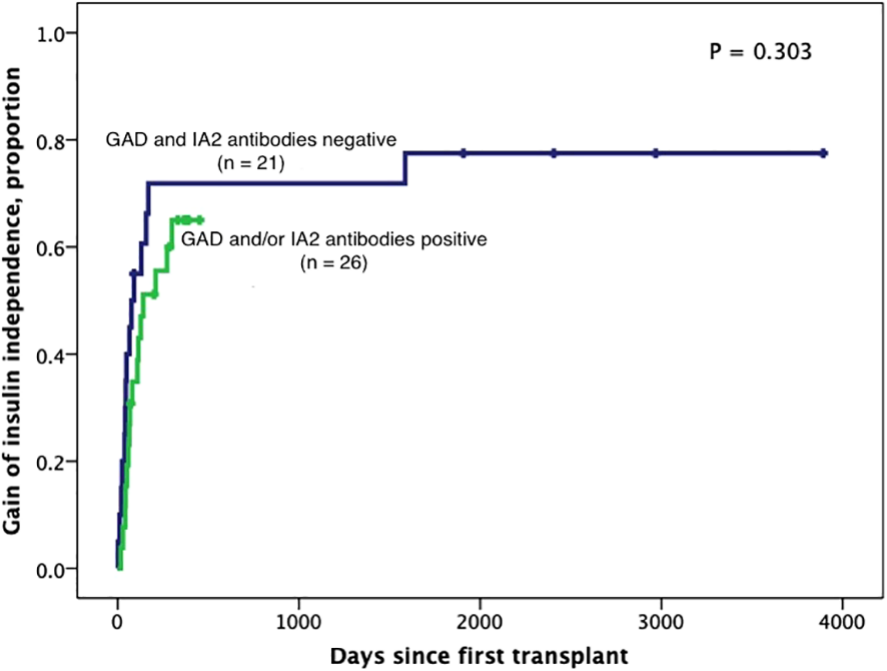
Kaplan-Meier curves of gain of insulin independence according to GAD65 and/or IA2 positivity (n=47). Patients were censored at the time they achieved insulin independence. Log-Rank test was performed for analysis between groups.

Subjects persistently positive had shorter allograft survival even after adjusting for induction immunosuppression (F [1,37] = 5.282; P = 0.027). We then individually analyzed GAD65 or IA-2 autoantibody positivity and their association with graft survival. Subjects with persistently single antibody positivity for GAD65 (n = 8) had shorter allograft survival compared to subjects persistently negative for both GAD65 and IA-2 (n = 21; P = 0.016, **Figure 2**). There was no difference in time of graft survival in subjects according to IA2 positivity only (P = 0.447; **Figure 3**).Gain of insulin independence was analyzed considering single GAD65 or IA-2 autoantibody positivity (**Figures 4**, **5**, respectively) or combined positivity (GAD65 and IA-2; **Figure 6**). Although the proportion of subjects achieving insulin independence was higher in subjects persistently autoantibody negative, results were not statistically significant (P = 0.303).

**Figure 7:**
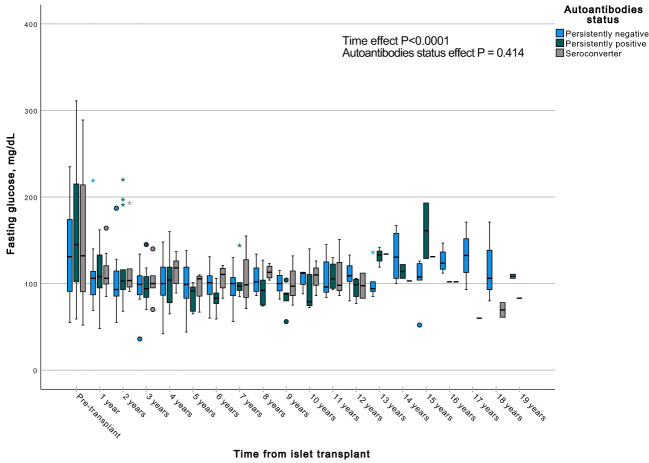
Box plots illustrating the variations in fasting glycemia across three groups (persistently negative, persistently positive, or seroconverters), demonstrating the temporal effect by comparing all time points with the pre-transplant period (P<0.0001). No significant impact of autoantibody status on fasting glycemia was observed (P=0.414), n=47. *Outliers.

**Figure 8:**
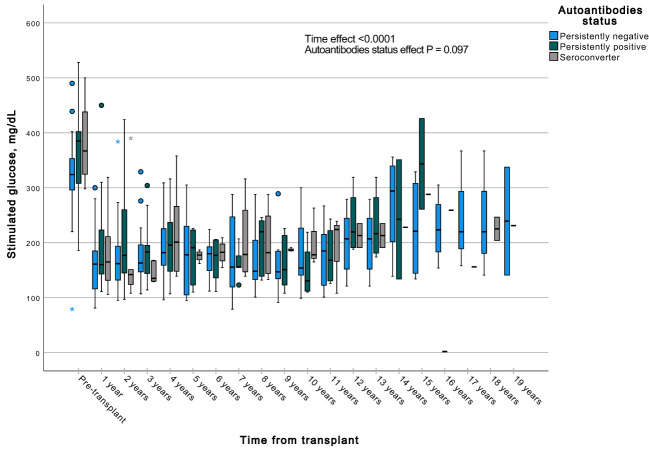
Box plots illustrating the variations in stimulated glycemia across three groups (persistently negative, persistently positive, or seroconverters), demonstrating the temporal effect by comparing all time points with the pre-transplant period (P<0.0001). No significant impact of autoantibody status on fasting glycemia was observed (P=0.097), n=47. *Outliers.

**Figure 9:**
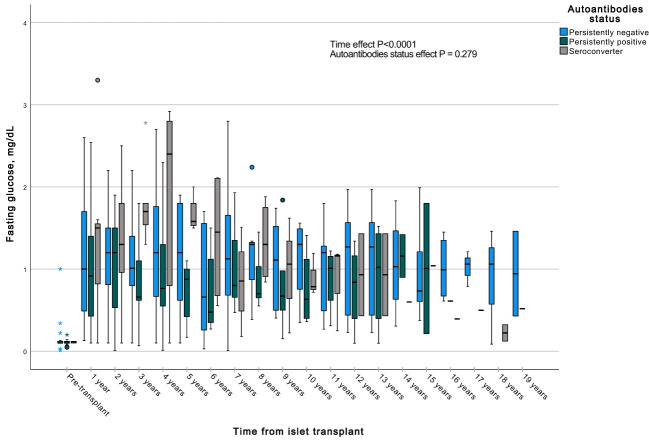
Box plots illustrating the variations in fasting c-peptide across three groups (persistently negative, persistently positive, or seroconverters), demonstrating the temporal effect by comparing all time points with the pre-transplant period (P<0.0001). No significant impact of autoantibody status on fasting glycemia was observed (P=0.279), n=47. *Outliers.

**Figure 10:**
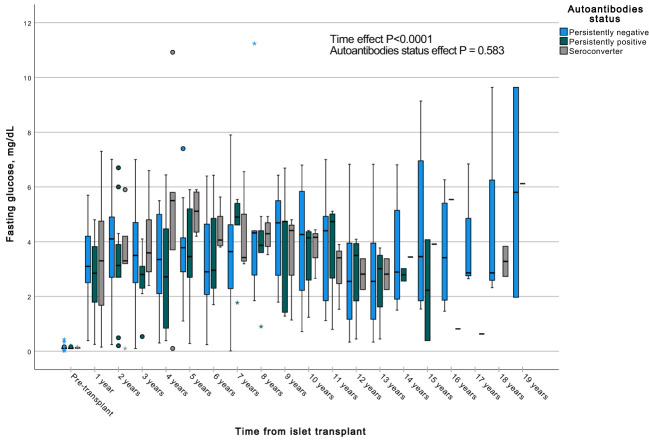
Box plots illustrating the variations in fasting glycemia across three groups (persistently negative, persistently positive, or seroconverters), demonstrating the temporal effect by comparing all time points with the pre-transplant period (P<0.0001). Notably, no significant impact of autoantibody status on fasting glycemia was observed (P=0.583), n=47. *Outliers.

A GEE with a gamma distribution was conducted to assess the impact of the presence of autoantibodies and time on a continuous measure of glucose and C-peptide, both during fasting and stimulated states in a MMTT in all subjects with graft function. We observed significant differences in all the measured parameters when comparing each time point to the pre-transplant period. Specifically, fasting and stimulated glycemia exhibited a significant decrease over time, up to 13 years post-transplant (**Figures 7**, **8**). In contrast, fasting and stimulated C-peptide levels displayed a significant increase throughout the entire duration of the study (**Figures 9**, **10**). However, there were no significant differences in these parameters among individuals with persistently negative, persistently positive, or seroconverter autoantibody statuses.

Lastly, we evaluated the frequency of HLA-DR3, -DR4, and -A24 positivity in the groups studied. Frequency of HLA-DR3 was 9.5% (n = 2) in persistently autoantibody negative subjects, 16.7 (n = 3) in persistently positive subjects, and none of the seroconverters was HLA-DR3 positive. Frequency of HLA-DR4 was 57.1% (n=12) in persistently autoantibody negative subjects, 55.6% (n=10) in persistently positive subjects, and 100% (n=8) in seroconverters. HLA-A24 alleles frequency was 14.3% (n=3), 0% and 25% (n=2) in subjects persistently negative, persistently positive, and seroconverters, respectively.

## Discussion

The main objective of the present study was to analyze the effect of GAD65 and/or IA-2 autoantibody positivity on islet allograft survival at our institution. Our findings revealed that persistent positivity of autoantibodies was associated with a decrease in the duration of islet allograft survival. Additionally, we observed that individuals who underwent seroconversion had a shorter graft survival compared to those who remained consistently negative for autoantibodies, although this difference did not reach statistical significance.

We have observed a lower percentage of positive serology for CMV in a cohort of 362 patients with type 1 DM, when compared with organ donors as representative of the general population (38% *vs* 67%) (18). This is not a result of a generalized unresponsiveness towards viral antigens, as the serological status of our study population to other common viruses (parvovirus and EBV) mimics the general population. Our observations suggest that a negative correlation between CMV serology and type 1 DM existed in the clinical population examined. The reasons for this significant difference from the general population is unclear. In our study, the percentage of CMV infection was similar among the groups. Therefore, there was no effect of CMV status on the influence 624 of autoantibodies on islet graft survival in the groups studied.

The existing scientific literature has conflicting findings regarding the influence of islet autoantibody status on the survival of islet allografts. Some studies indicate that the presence of islet autoantibodies before transplantation is not linked to islet allograft survival (19–21). Conversely, another study suggests that pre-transplant autoantibody positivity is indeed associated with the survival of islet allografts (22). Furthermore, it has been demonstrated that the development of autoantibodies after islet transplantation, as well as an increase in antibody titers, can predict the failure of islet transplants (19, 20). On the other hand, Anteby et al. (21) found no association between GAD-65 antibody positivity and islet allograft outcomes, regardless of whether the positivity persisted or appeared following islet transplantation. It should be noted, however, that the cohort in this particular islet transplant study lacked IA2 and ZnT8 autoantibodies pre- and post-transplantation, therefore the impact of these islet autoantibodies on allograft survival could not be evaluated (21).

The immunosuppressive therapies currently used for maintenance purposes are highly effective in preventing rejection of transplanted islets. However, they may not fully prevent the development of islet autoantibodies. Nonetheless, these therapies could potentially delay the onset of autoimmune-related failure of the transplanted islets. It has been observed that the autoantibody positivity can occur several years prior to the recurrence of T1D following a pancreas transplant (12).

It is important to emphasize that a considerable number of previous studies were carried out before the establishment of the Edmonton Protocol era (16). Consequently, individuals with T1D in those studies were administered islets using less potent immunosuppressive protocols. These variations in immunosuppressive regimens are likely responsible for the inconsistencies observed in the impact of autoantibodies on the outcomes of islet transplantation among different studies. Furthermore, other factors that contribute to these inconsistencies include variations in patient characteristics, duration of follow-up, quality of islet preparation, and the specific surgical procedures associated with the transplantation process.

In our patient cohort, we noted that all seroconverters were HLA-DR4 positive suggesting that these subjects have a heightened immune response to islet antigens. This may be an important observation as it may suggest that HLA-DR4 positive subjects may be more susceptible to immune recognition of islet antigens and reactivation of islet autoimmunity when re-exposed to pancreatic β-cells from an islet allograft.

Individuals who carry HLA-DR3 or HLA-DR4 alleles have a higher risk of developing T1D, as these alleles are involved in the recognition and destruction of β -cells by T cells (23, 24).

In the present study, the frequency of HLA-DR3 and HLA-DR4 was not associated with the status of islet autoantibodies (negative, positive, or converter). However, interestingly, 100% (n = 8) of seroconverters carried the HLA-DR4 allele. Furthermore seven out of the 8 seroconverters underwent induction with anti-IL2 antibodies. Whether induction with a T-cell depletion regimen may have impacted islet autoimmunity differently in the seroconverter group remains unclear.

A study by Demeester et al. (25) showed that islet transplant recipients carrying the HLA-A24 allele were at higher risk of developing autoantibodies post-transplantation and a faster decline in islet graft function compared to non-carriers. However, we did not find an association between the presence of HLA-A24 alleles and islet autoantibody status in our study cohort.

In the present study, the number of patients who developed alloantibodies, accompanied by the appearance of HLA class I–or class II–donor specific antibodies, was very small, suggesting that autoreactivity, as indicated by persistence and/or *de-novo* formation of islet autoantibodies, may be independent of allo-rejection. Therefore, the effect of alloantibodies on islet allograft survival was not included as a covariate in our multivariable analysis.

We also observed that several of our patients converted to positive autoantibodies after the discontinuation of immunosuppression (data not shown). This finding supports that immunosupresion may avoid islet auto and allo-reactivity (26).

The ideal biomarker of autoimmunity recurrence would be, in addition to the autoantibodies, the finding of circulating islet autoantigen-specific T-cell clones (CD4 and CD8) in the peripheral blood, preceding the loss of c-peptide in islet transplant recipients, but there are several limitation to these assays and they are not widely available (27).

In T1D recipients of SPK, despite immunosupression, recurrence of T1D has been demonstrated by onset of hyperglycemia in the setting of islet autoantibodies seroconversion, in addition to identification of circulating pathogenic autoreactive CD4 T-cells and histology proven insulitis and β-cell loss, in the absence of allo-rejection (12).

In pancreas transplantation, Ringers et al. described that the incidence of rejection episodes was higher in recipients who were GAD-65 antibody positive and treated with Daclizumab compared to GAD65 negative recipients or ATG-treated recipients, suggesting that the pre-transplantation positivity of GAD65 autoantibodies may be a factor to be considered for the selection of immunosuppression induction in this population (28). Hilbrands et al., in this context suggest that islet autoantibody positive subjects should receive T-cell depletion as immunosuppressive induction for islet transplantation (29).

Most of the patients in our study cohort received the Edmonton protocol as the immunosuppressive induction, instead of a T-cell depletion regimen. In our analyses, the use of T-cell depletion did not influence the effect of autoantibody positivity on graft survival. However, a larger prospective cohort study is needed to further address this issue.

With regards to the impact of islet autoantibody status on insulin independence following islet transplantation, some studies have suggested that islet autoantibodies interfere with achievement of insulin independence (29, 30) whereas in others, islet autoantibodies did not significantly alter the proportion of subjects achieving insulin independence (3, 31). In our study, attainment of insulin independence was not affected by persistently single or double autoantibody positivity (GAD65 or IA2).

It is important to acknowledge the limitations of our study. Firstly, we did not assess pre-transplant cellular autoreactivity, which has been previously described by Hurmann (3). Additionally, the absence of other autoantibodies, such as ZnT8 antibodies, restricts the breadth of our study conclusions. In cases of solitary pancreas transplantation, incorporating the assessment of relative changes in ZnT8 antibody levels alongside changes in established antibodies, following the transplant procedure, can significantly improve the ability to predict the long-term survival of the graft. This improvement is evident through the increased sensitivity, specificity, and predictive value (32). When ZnT8 is detected in pancreas transplant recipients, its appearance is short-lived, compared to GAD65 and IA2 antibodies, and precedes the development of hyperglycemia with T1D recurrence, suggesting a short but important role (33). Similarly, in the field of islet cell transplantation, the addition of ZnT8 to GAD65 and IA-2 antibodies in the screening panel, increases the ability to identify those individuals at risk for poor islet graft outcomes (19).

On the other hand, our study benefits from a relatively large group of individuals with T1D (47 subjects) who underwent islet transplantation. A notable strength of our study is the frequent sampling conducted after the transplantation procedure. This frequent sampling helps to reduce the likelihood of misclassifying individuals’ autoantibody status as false positive or false negative due to the inherent fluctuations of circulating autoantibodies over time.

In conclusion, islet transplant recipients with persistent positivity for GAD65 and/or IA2 autoantibodies had reduced allograft survival, independent of immunosuppressive treatment. Islet autoantibody positivity to GAD65 and/or IA2 did not interfere with attainment of insulin independence after islet allograft transplantation. Despite adequate immunosuppression, our data suggest that islet autoimmunity remains a factor contributing to islet allograft failure.

Larger prospective studies are needed to further address the role of islet autoantibody status on islet graft survival.

## Data availability statement

The raw data supporting the conclusions of this article will be made available by the authors, without undue reservation.

## Ethics statement

The studies involving humans were approved by The University of Miami Institutional Review Boards. The studies were conducted in accordance with the local legislation and institutional requirements. The participants provided their written informed consent to participate in this study.

## Author contributions

JL: Data curation, Formal Analysis, Writing – original draft, Writing – review & editing. RP: Writing – original draft, Writing – review & editing. JA: Data curation, Writing – original draft. NB: Data curation, Writing – original draft. AA: Data curation, Writing – review & editing. NP: Writing - review & editing, Data curation. FV: Data curation, Methodology, Writing – review & editing. CR: Funding acquisition, Resources, Supervision, Writing – review & editing. DB: Investigation, Methodology, Project administration, Writing – original draft, Writing – review & editing. RA: Investigation, Writing – original draft, Writing – review & editing, Conceptualization, Funding acquisition, Resources, Supervision.
